# Lung Metastasis From Chronic Lymphocytic Leukemia

**DOI:** 10.1016/j.atssr.2025.12.021

**Published:** 2026-01-17

**Authors:** Mikenzie Sturdevant, Ryan Brownlee, Mark Dalgetty, Natashia Savage, Girindra Raval, Locke Bryan, Daniel Miller

**Affiliations:** 1 Department of Surgery, Medical College of Georgia, Augusta University, Augusta, Georgia; 2 Medical College of Georgia, Augusta University, Augusta, Georgia; 3 Division of Hematology and Oncology, Department of Medicine, Medical College of Georgia, Augusta, Georgia

## Abstract

Chronic lymphocytic leukemia (CLL) is a mature B-cell neoplasm diagnosed through blood counts, differential counts, blood smear, and immunophenotyping, and it can have a highly variable clinical presentation and course. In a 66-year-old man with a history of CLL, multiple pulmonary nodules were identified on a routine low-dose computed tomographic scan for lung cancer screening for his smoking history. Follow-up positron emission tomography with computed tomography showed indeterminate, low-level avidity nodules, prompting a right lower lobe wedge resection by video-assisted thoracoscopy for tissue diagnosis. Histologic examination displayed a sharply demarcated nodular infiltrate of small lymphocytes with coarse chromatin. Flow cytometry revealed a kappa light-chain–restricted CD19^+^ population of B cells coexpressing CD5 and CD23. Staining showed negative cyclin D1. This represents a rare case of CLL with lung metastasis.

Chronic lymphocytic leukemia (CLL) is an accumulation of dysfunctional mature B lymphocytes displaying impaired apoptosis, most often driven by BCL2 overexpression and constitutionally activated B-cell receptor signaling.^1^ Persistent lymphocytosis is the most common presentation and often prompts further evaluation. Subsequent flow cytometry testing is the primary tool used for establishing a CLL diagnosis, by reliably distinguishing it from other similar B-cell lymphoproliferative disorders.^1^

Approximately 70% of patients are asymptomatic, leading to incidental diagnosis as a result of lymphocytosis or pathologic examination obtained for unrelated medical reasons. Rarely, CLL is identified incidentally within the lungs, such as during an isolated lung transplantation case described in a 2021 case study.^2^ Because lung cancer is the leading cause of cancer-related mortality in the United States and given its commonly late-stage diagnosis, in 2013 the US Preventive Services Task Force recommended lung cancer screening in high-risk individuals with an annual low-dose computed tomographic (CT) scan of the chest.^3^ High-risk individuals are defined as patients aged more than 50 years, persons with more than a 20-pack-year smoking history, and smokers who have not quit within the last 15 years.^3^ Once an indeterminate nodule <3 cm is identified on screening, management is guided by the nodule size, clinical risk of malignancy, patient preferences, and overall health status.^4^

A 66-year-old man, with a significant history of CLL and a 40-pack-year smoking history, was found to have multiple, bilateral pulmonary nodules on a low-dose CT chest scan that was obtained for lung cancer screening (Figure 1). Two years previously, he was noted to have a paranasal growth, which was resected and showed monotypic B cells positive for CD5, CD20, and CD23. His peripheral blood flow cytometry showed a monotypic B-cell population that was CD5 and CD20 positive. Results of testing for BCL1 were negative, without high-risk cytogenetics on fluorescence in situ hybridization on staining. He was followed up every 3 to 6 months with blood testing but never had any B symptoms and so remained on active surveillance.

**Figure 1:**
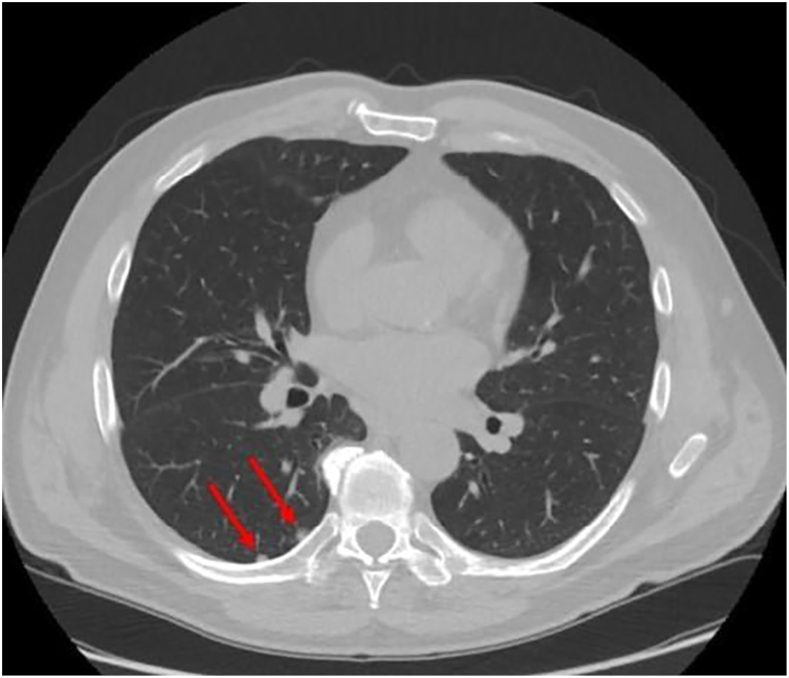
Screening chest computed tomography. Axial image demonstrates 2 subpleural right lower lobe nodules (arrows).

The CT chest scan was followed up with a fluorine-18 fluorodeoxyglucose positron emission tomographic (PET)–CT scan, which showed low-level avidity of mediastinal and abdominal lymph nodes consistent with Deauville score 3. All of the lung nodules remained indeterminate and were below PET resolution. Given the indeterminate findings, the patient was referred to a thoracic surgeon to obtain a tissue diagnosis. The thoracic team proceeded with right-sided video-assisted thoracoscopic surgery, and the nodules were identified through palpation of the posterior aspect of the right lower lobe. Because of their close proximity to each other, both nodules were collected in a single wedge resection. Lymphoid infiltrate was identified on frozen section. The second nodule was sent for lymphoma protocol for definitive classification.

Grossly, the mass was distinct, nodular, and well demarcated (Figure 2A). Permanent sections showed pulmonary parenchyma with a nodular infiltrate composed of small lymphocytes with coarse chromatin (Figures 2B, 2C). No granulomas, malignant nonhematopoietic cells, or significant numbers of large lymphocytes were seen. A cyclin D1 immunohistochemistry stain was performed, and the result was negative. Flow cytometry phenotyping of the mass revealed a CD19 B-cell population with overt kappa restriction, aberrant coexpression of CD5 and CD23, and aberrant downregulation of CD20 (Figure 3). The morphologic and immunophenotypic features of these nodules were consistent with CLL.

**Figure 2:**
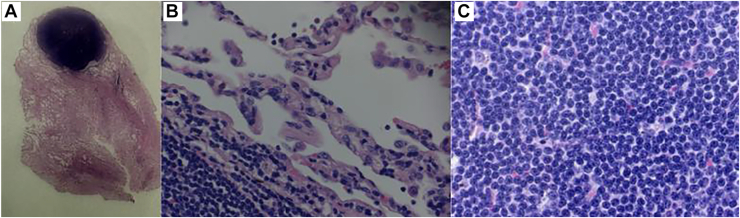
Pulmonary wedge resection showing chronic lymphocytic leukemia. (A) Low-power view wedge resection specimen demonstrating a sharply demarcated mass. (Hematoxylin and eosin; original magnification ×10). (B) Mass showing small lymphocytes. (Hematoxylin and eosin; original magnification ×200.) (C) Mass revealing infiltrate of discohesive, monotonous, small lymphocytes with coarse chromatin and smooth nuclear contours. (Hematoxylin and eosin; original magnification ×400.)

**Figure 3:**
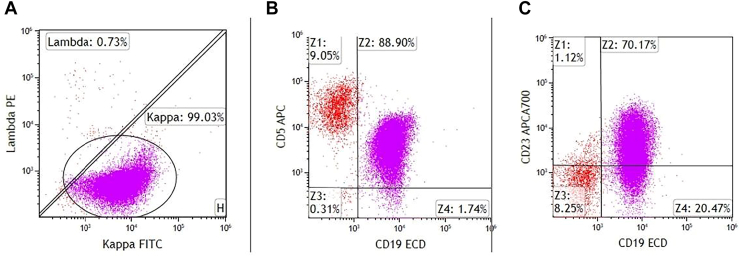
Flow cytometric immunophenotyping of the pulmonary mass. (A) B-cell overt kappa restriction. (B) Aberrant CD5 expression. (C) Aberrant CD23 expression. (APC, adenomatous polyposis coli gene; ECD, Erdheim-Chester disease; FITC, fluorescein isothiocyanate; PE, pulmonary embolism)

The patient continued to deny B symptoms, including no fever, night sweats, or unintentional weight loss. Imaging demonstrated no bulky lymphadenopathy, and his laboratory values showed no signs of progressive anemia, progressive thrombocytopenia, significant cytopenia, and stable white blood cells at approximately 20 × 10^3^/μL with an absolute lymphocyte count of 17 × 10^3^/μL. The findings were further discussed with the patient, and a decision was made to continue active surveillance with repeat laboratory testing in 3 months.

## Comment

This patient’s presentation with multiple, bilateral nodules located along the pleural surface, the largest of which measured 7.4 mm, led to an indeterminate interpretation. Nodules of <8 mm and >5mm have an intermediate lung cancer probability of approximately 1%, using data from the NELSON trial.^4^^,^^5^ Moreover, the location of the nodules on the pleural surface was more consistent with benign features.^4^ Given these findings, we obtained the PET-CT scan before proceeding with tissue diagnosis, which continued to be indeterminate. After shared decision making and considering his history of smoking and CLL, we decided to proceed with resection for diagnosis rather than biopsy because of the very peripheral location of the nodules.

CLL with pulmonic leukemic infiltration is a rare pathologic process that has been reported only in a small subset of patients. It has been documented more commonly on autopsy, but it is very rare to see radiographically evident mass-forming lung nodules.^6^ In patients with CLL with lung nodules, it is not advised to rely solely on PET-CT imaging because it leaves uncertainty regarding the origin. However, PET-CT scanning has been shown to be effective in detecting Richter transformation.^7^ If intraoperative frozen sections show small B-cell infiltrates, flow cytometry and immune phenotyping should follow to distinguish CLL from other lymphoproliferative disorders.

In this report we demonstrate that small, low-fluorodeoxyglucose nodules in patients with CLL may indicate nonprimary lung involvement that can be diagnosed safely with lung wedge resection, flow cytometry, and immunohistochemistry.
